# High phosphoserine in sepsis: panel of clinical and plasma amino acid correlations

**DOI:** 10.1186/2193-1801-3-279

**Published:** 2014-06-02

**Authors:** Carlo Chiarla, Ivo Giovannini, John H Siegel

**Affiliations:** CNR-IASI Center for the Pathophysiology of Shock, Department of Surgical Sciences, Catholic University of the Sacred Heart School of Medicine, Largo A. Gemelli 8, I-00168 Rome, Italy; New Jersey Medical School of Rutgers University, Newark, USA

**Keywords:** Plasma phosphoserine, Sepsis, Critical illness, Proteolysis, Phosphoproteomics, Organ failure

## Abstract

The determination of plasma phosphoserine concentration in sepsis is uncommon, and the clinical and metabolic correlations of abnormally high phosphoserine are basically unknown. We analyzed 430 determinations of phosphoserine, other amino acid (AA) levels and ancillary variables obtained in 18 septic patients, in order to assess the biochemical and clinical correlations of changes in phosphoserine. Phosphoserine ranged between 5 and 55 micromol/L (n.v. < 12). Increasing phosphoserine was associated with higher severity of illness and death, and its best AA correlates were increasing cystathionine, 3-methylhistidine, histidine, hydroxyproline and tyrosine (r > 0.65, p < 0.001 for all). High phosphoserine seemed to cumulatively reflect kidney and liver dysfunction and enhanced proteolysis. As a collateral finding, 3-methylhistidine (a best correlate of phosphoserine) was also inversely related to nutritional AA dose (p < 0.001). These data suggest that in septic patients the determination of plasma phosphoserine may provide useful information on severity of septic illness and prognosis. The observed correlations also indirectly evidenced an impact of nutritional AA dose in moderating proteolysis.

## Background

No adequate information exists regarding the patterns of changes of plasma phosphoserine in sepsis, and the related clinical implications. Although high phosphoserine has occasionally been reported in the past as a feature of lethal septic illness (Roth et al. 1982), its relationship with changes in other amino acid (AA) and biochemical variables, and with the associated clinical patterns, has not been sufficiently explored. Even the correlations between phosphoserine, its biochemical byproduct serine and the closely related AA glycine (Wu 2009; Bender 2012; Wang et al. 2013) were never systematically assessed, at least to our knowledge. We have inconsistently observed, in septic patients, remarkable increases in phosphoserine, and the aim of the present study was to better characterize the patterns of changes in phosphoserine in sepsis, by quantifying in detail the correlations with other AAs, and with the simultaneously observed alterations of blood chemistries and clinical variables.

## Methods

Data from 430 plasma AA assessments performed in 18 septic patients, with the corresponding metabolic and clinical variables recorded in our patient AA database, were analyzed in detail. The patients (4 women, 14 men) developed sepsis after trauma with a combination of abdominal, chest and head injuries. Their median age was 25.5 years (range 16–71), weight was 75 Kg (40–120), and height 174 cm (163–188). The cause of sepsis was intra-abdominal, pulmonary or extensive soft tissue infection. The diagnosis of sepsis was based on the occurrence of a temperature > 38.3°C, white blood cell count > 12 × 10^9^/L or < 3 × 10^9^/L and clear evidence of infection verified by positive cultures from blood, surgical drainage of infected areas, or sputum in the case of pulmonary sepsis. Median sepsis severity score (Skau 1985) upon diagnosis of sepsis was 24 (range 11–75). Most patients survived, some of them after evolving through near-fatal illness, two patients died from septic metabolic and cardiorespiratory decompensation from multiple organ dysfunction, and one suffered sudden death without multiple organ dysfunction. The AA assessments were performed every 8 to 24 hours until the clinical criteria for the diagnosis of sepsis persisted, for a total of 430 measurements. For this purpose, arterial blood samples were collected into EDTA vacutainer tubes and the plasma was immediately separated and deproteinized with a sulfosalicylic acid solution. The AA analyses were performed on the fresh plasma samples, or the plasma was frozen and stored at −20°C and the assay was performed within one week on a Beckman 6300 high-performance AA analyzer (Beckman Instruments, Inc., Palo Alto, CA). Phosphoserine was included in the AA profile. The Fischer AA ratio (Freund et al. 1979) was calculated according to the ratio (leucine + isoleucine + valine)/(phenylalanine + tyrosine). In many instances serum creatinine, bilirubin and ammonia were simultaneously available. The Sepsis-related Organ Failure Assessment score (SOFA score) (Vincent 2006) was additionally determined. The patients were receiving total parenteral nutrition (34 ± 14 kcal/kg/24 hr, about three fourths glucose and one fourth fat, and 1.5 ± 0.6 g/kg/24 hr amino acids). To account for possible transient variations in infusion rate, the infusion rates maintained in the last eight hours before blood sampling were recorded and used for the study. The study protocol complied with the 1964 Helsinki declaration. It consisted of the secondary analysis of a previously developed patient AA database, and patients or their relatives gave their consent to take part in the original study Medians and ranges or 5^th^ - 95^th^ percentiles were used as indices of centrality and dispersion of the distributions. The measurements provided a continuous distribution of observations over a wide range of conditions, extending from moderately to extremely severe septic illness. This was well suited to assessing the correlations of phosphoserine over an ample area of pathophysiologic abnormalities. The relationships existing between phosphoserine and the other AAs and the metabolic variables were explored on two- or three-dimensional graphical displays. Further assessments and validations of the results were performed by Student’s t test, Mann–Whitney–Wilcoxon test and by least-square regression and covariance analysis, and analyzed for the Pearson correlation coefficient, with skewness and kurtosis control, and analysis of residuals (Statgraphics Plus, Manugistics, Rockville, MD). To specifically address only the best correlates of phosphoserine, correlations with absolute r < 0.30 were not taken into account. Significance of covariance was assessed by Scheffé criteria (based on confidence intervals and differences in slope and intercept) (Seber 1977) and with the selection of the simplest possible regressions yielding the best control of variability of phosphoserine.

## Results

Values of plasma AAs are shown in Table 1. Plasma phosphoserine ranged between 5 and 55 micromol/L (median 12; normal value < 12). By evaluating patient trends, increasing phosphoserine soon appeared to be generally associated with increasing severity of illness. In the two patients who died from multiple organ dysfunction phosphoserine was 25.6 ± 16.8 (mean ± SD; median 15.5, range 11–55) while in survivors it was 12.6 ± 4.4 (median 12.0, range 5–41) (p < 0.001). The correlations with other AAs were assessed by regression analysis.

**Table 1:** **Medians and 5** ^**th**^ **-95** ^**th**^ **percentiles for plasma AAs**

Phosphoserine	12 (7–22)
Phosphoserine/serine	0.11 (0.06-0.28)
Cystathionine	4 (1–18)
3-methylhistidine	5 (1–18)
Histidine	72 (52–240)
Hydroxyproline	11 (6–31)
Tyrosine	57 (38–114)
Serine	103 (55–179)
Glycine	239 (140–578)
Threonine	106 (58–293)
Alanine	281.5 (174–675)
Phenylalanine	116 (78–226)
Glutamine	449.5 (323–1014)
Cystine	47 (27–80)
Proline	188 (99–965)
Alpha-amino-n-butyric acid	13 (4–59)
Arginine	92 (49–186)
Asparagine	40 (22–210)
Aspartic acid	7 (2–26)
Citrulline	11 (6–43)
Glutamic acid	59 (16–188)
Isoleucine	76 (35–153)
Leucine	128.5 (82–233)
Lysine	170.5 (99–327)
Methionine	41 (20–180)
Ornithine	73 (40–188)
Phosphoethanolamine	9 (3–28)
Taurine	73 (22–242)
Tryptophan	55 (33–78)
Valine	256.5 (156–489)
Fischer ratio	2.4 (1.52-5.75)

### General correlations

Regression analysis showed that, among the neutral AAs, phosphoserine was directly correlated to glycine (r = 0.60), threonine (0.57), alanine (0.51) and serine (0.50) (p < 0.001 for all) without relevant correlations with leucine, isoleucine or valine. Among the acidic AAs and amides, phosphoserine was directly correlated to glutamine (r = 0.57, p < 0.001) and unrelated to glutamate or aspartate. There were no relevant correlations with the basic AAs arginine, lysine and ornithine. Among the aromatic AAs, phosphoserine was directly related to histidine (r = 0.71), tyrosine (0.66) and phenylalanine (0.57) (p < 0 .001 for all), and unrelated to tryptophan. Among the sulphur AAs, phosphoserine was directly related to cystathionine (r = 0.88) and cystine (0.55) (p < 0.001 for both), and unrelated to methionine and taurine. Phosphoserine was also directly related to the cyclic AA proline (r = 0.51) and, among less commonly considered AAs, it was directly related to 3-methylhistidine (r = 0.71), hydroxyproline (0.69) and alpha-amino-n-butyric acid (0.60) (p < 0.001 for all), and was unrelated to phosphoethanolamine. Finally phosphoserine was inversely related to the Fischer AA ratio (r = −0.32) and, among non-AA variables, it was directly related to SOFA score (r = 0.55), creatinine (0.49), bilirubin (0.42) and ammonia (0.31) (p < 0.001 for all). Creatinine, bilirubin and ammonia together accounted for 45% of the variability of phosphoserine (multiple r = 0.67, r^2^ = 0.45, p < 0.001).

### Correlations between phosphoserine, serine, and glycine

As already mentioned, phosphoserine was directly related to its biochemical byproduct serine (r = 0.50) and to the companion AA glycine (r = 0.60, p < 0.001 for both). In turn, serine and glycine were directly and tightly interrelated (r = 0.79, p < 0.001). More detailed analysis of their balance showed the maintenance of lower serine for any given glycine level in the presence of increasing phosphoserine (p < 0.001) and, by considering the other available measurements, also in the presence of increasing 3-methylhistidine, creatinine or bilirubin (p < 0.001 for all). Similarly, the phosphoserine/serine ratio significantly increased with increasing creatinine, bilirubin and SOFA score (p < 0.001 for all), however, contrary to the absolute phosphoserine value, the phosphoserine/serine ratio was not significantly higher in nonsurvivors compared to survivors.

### Best correlates of phosphoserine

Therefore the best AA correlates of increasing phosphoserine were cystathionine, 3-methylhistidine, histidine, hydroxyproline and tyrosine (while other correlations seemed to mostly reflect metabolic affinities among various AAs, and a general tendency for hyperaminoacidemia in worse stages of illness). In nonsurviving patients, and in survivors during more severe illness at peaking SOFA score, phosphoserine increased evidently together with the listed AAs. However the increase in phosphoserine was comparably greater, which resulted in higher phosphoserine level for any given level of cystathionine, 3-methylhistidine, histidine, hydroxyproline and tyrosine. This was reconfirmed by assessing values of these AAs for cases with phosphoserine > 12 micromol/L (median value) compared to cases with phosphoserine ≤ 12 (p < 0.001 for all, Table 2).

**Table 2:** **Plasma phosphoserine correlates**

	Phosphoserine ≤ 12	Phosphoserine > 12
Phosphoserine	10 (7–12)	15 (13–27)
Cystathionine	3 (1–13)	6 (1–27)
3-methylhistidine	3 (1–7)	10 (2–29)
Histidine	69.5 (50.5-97)	80 (54–409)
Hydroxyproline	10 (5–22)	14 (6–57)
Tyrosine	54 (36–91)	59 (40–166)
SOFA score	6 (3–10)	10 (3–15)
*p < 0.001 for all differences*		
**Regression equation:**		
3-methylhistidine = 4.32 + 0.45 (phosphoserine) +0.03 (creatinine) –3.92(AA dose)		
*multiple r = 0.80; partial r for AA dose = − 0.44; p < 0.001 for each coefficient and for whole regression*		

**Upper part.** Medians and 5^th^ - 95^th^ percentiles for the best AA correlates of phosphoserine (micromol/L for all) and SOFA score, for measurements with phosphoserine > 12 (median value) compared to measurements with phosphoserine ≤ 12. **Lower part**. Regression equation assessing the relationship between plasma 3-methylhistidine, phosphoserine, creatinine (micromol/L for all) and nutritional AA dose (g/kg/day). Median creatinine 70.72; 5^th^ - 95^th^ percentiles 35.36 - 238.68.

Detailed analysis of the correlation between phosphoserine and 3-methylhistidine showed that this was influenced by creatinine level (to which both AAs were directly related, p < 0.001 for both), and that only 3-methylhistidine was simultaneously and inversely related to the nutritional AA dose (r = −0.44, p < 0.001), as shown in the multiple regression in Table 2. Three-methylhistidine was unrelated to other substrate doses. Cystathionine, histidine and hydroxyproline were only weakly related to creatinine (r < 0.27, p < 0.001 for all) and, similarly to phosphoserine, were unrelated to nutritional substrate doses.

## Discussion

Phosphoserine is an intermediate in the production of serine through the glycolytic pathway: 3-phosphoglycerate is first oxidized to form 3-phosphohydroxypyruvate, which is then transaminated to form phosphoserine. Subsequently, through the action of the enzyme phosphoserine phosphatase, phosphoserine is converted to serine (de Koning et al. 2003; Bender 2012). Plasma serine is then involved in a tight balance with glycine, and the two AAs are interconvertible, which is in agreement with the close correlation found in our study between serine and glycine (r = 0.79, p < 0.001).

A different issue regards the phosphoserine which is formed within proteins as the result of reversible post-translational phosphorylation of their serine residues. This is among the fundamental processes regulating protein function, is investigated in the field of phosphoproteomics and may in itself be involved in the metabolic abnormalities of sepsis (Sickmann and Meyer 2001; Ubersax and Ferrell 2007; Wu 2009; Chen et al. 2011).

Increases in plasma phosphoserine have generally been associated with pyridoxal-5-phosphate (vitamin B_6_) and magnesium deficiency (Lord and Bralley 2008). Apart from this, no other implications have been described, at least to our knowledge, although phosphoserine may easily be measured together with the other AAs, and although higher phosphoserine was occasionally reported in lethal versus non-lethal sepsis and acute necrotizing pancreatitis, without exploring the involved correlations (Roth et al. 1982; Roth et al. 1985).

Our study showed that the best AA correlates of increasing phosphoserine in sepsis were increasing cystathionine, 3-methylhistidine, histidine, hydroxyproline and tyrosine (r > 0.65, p < 0.001 for all). Less tight correlations were mostly reflecting metabolic affinities among these and other AAs (for instance affinities involving the neutral AAs glycine, serine and threonine, or the aromatic AAs tyrosine and phenylalanine), and a general tendency for hyperaminoacidemia in worse stages of illness.

Increasing cystathionine is known to be associated with both kidney and liver dysfunction, and impairment of hepatic AA transsulfuration is likely involved in the latter (Look et al. 2000). Increases in the aromatic AAs tyrosine and phenylalanine also represent consequences of liver dysfunction, as does the decrease in Fischer AA ratio (cumulatively accounting for hepatic-mediated imbalances in branched chain and aromatic AAs) (Freund et al. 1979) which also correlated with increasing phosphoserine.

Increasing plasma 3-methylhistidine is an index of proteolysis (mainly of myofibrillar proteins) (Hasselgren and Fischer 1998); furthermore, this post-translationally methylated histidine is excreted by the kidney, therefore its level is also expected to correlate with renal dysfunction and creatinine, as demonstrated by the multiple regression in Table 2. Of note, in our study, in spite of the heterogeneous patient conditions, nutritional AA dose emerged as another important determinant of 3-methylhistidine, however associated with its decrease.

Increasing hydroxyproline has a meaning similar to that of 3-methylhistidine because the post-translational hydroxylation of proline in proteins produces hydroxyproline, and hypercatabolism (mainly connective tissue degradation) enhances release into plasma and urinary excretion of hydroxyproline (Beisel 1986; Gäddnäs et al. 2009; Wu et al. 2011).

Therefore the AA correlations in our study suggest that in sepsis elevations of phosphoserine may cumulatively reflect abnormal kidney and/or liver dysfunction and enhanced proteolysis, and thus degree of illness. This was reconfirmed by the direct correlations found between phosphoserine and creatinine, bilirubin and ammonia, which together accounted for 45% of the variability of phosphoserine (multiple r = 0.67, r^2^ = 0.45), and by the correlation with the SOFA score (r = 0.55, p < 0.001). The correlation with histidine could less easily be explained, even though high histidine may reflect the enhanced sum of endogenous protein and carnosine (beta-alanyl-histidine) breakdown (Enwonwu et al. 2000).

In evaluating the association of increasing phosphoserine and of its best AA correlates with severity of illness, there is to observe that a relevant source of phosphoserine may be the breakdown of phosphorylated proteins and cell phospholipids (Rossi et al. 1980; Lord and Bralley 2008). Although we cannot quantify its contribution, a characteristic shared by phosphoserine, 3-methylhistidine and hydroxyproline is that all three AAs derive from post-synthetic modifications of proteins (Bender 2012), and enhanced proteolysis could partly explain the tendency for parallel increases of these AAs, while common dependency on kidney dysfunction should be an additional factor. Phosphoserine had a major relevance in our patients because, during worsening of illness, its increase was comparably greater than that of 3-methylhistidine and hydroxyproline. As already mentioned, plasma phosphoserine may in large part derive from the catabolism of proteins and phospholipids (Rossi et al. 1980; Lord and Bralley 2008). Intracellular synthesis of phosphoserine occurs in the glycolytic pathway, in which conversion of 3-phosphoglycerate into 3-phosphohydroxypyruvate is catalysed by 3-phosphoglycerate dehydrogenase, and the latter is subsequently converted into phosphoserine (3-phosphoserine) by 3-phosphoserine aminotransferase (van der Crabben et al. 2013). Phosphoserine may be finally converted into serine (L-serine) by phosphoserine phosphatase, while phosphorylation of serine can only take place when it is a component of proteins (Rossi et al. 1980). In theory, expansion of the phosphoserine pool could derive from enhanced septic glycolytic flux, from enhanced protein and phospholipid catabolic drive, and from kidney dysfunction if present. On speculative grounds, also insufficient activity of phosphoserine phosphatase should be considered. Our data cannot account for all these possibilities. However the assessed correlations support a major role of protein catabolism. Phosphoserine is the most abundant AA among the phosphorylated AA residues in proteins, including human skeletal muscle and liver proteins (Olsen et al. 2006; Ubersax and Ferrell 2007; Højlund et al. 2009; Lundby et al. 2012; Song et al. 2012), phosphoserine outflow from muscle in injury was already demonstrated in the past (Brooks et al. 1986), and this is in agreement with the strong correlation that we found between phosphoserine and 3-methylhistidine.

Other findings in our study regarded the balance between phosphoserine and the biochemically related AAs serine and glycine. The phosphoserine/serine ratio behaved similar to phosphoserine, but showed comparably smaller increases. Increasing phosphoserine and other signs of worsening of illness were associated with maintenance of lower serine for any given glycine level; although this was in agreement with previous findings on renal insufficiency (Bender 2012; Fürst and Stehle 2004) the clinical relevance for our patients was uncertain. Conversely, very relevant appeared the ancillary finding of a significant impact of nutritional AA support in decreasing 3-methylhistidine for any given creatinine and phosphoserine level (multiple regression in Table 2), and therefore in moderating proteolysis. This was not new in itself, however it was impressive that nutritional AA dose emerged so strongly (partial r = −0.44, p < 0.001) as a likely determinant of reduced proteolysis, in spite of the multiple causes of inter-patient variability, further reaffirming the important role of nutrition in sepsis.

## Conclusion

In human sepsis there is an unmet need for additional biomarkers of severity of illness, to improve decision making for high-risk patients. Furthermore, in the field of metabolomics, the awareness of new biomarkers may generate more efficient metabolomic signatures for clinical purposes. The determination of plasma phosphoserine is uncommon and inconsistently included in reported AA-grams, and the biochemical and clinical correlations of this AA are imperfectly known. Our data and the observed correlations suggest that in septic patients the determination of phosphoserine, or its regular inclusion in AA-grams and other multi-analyte profiling methodologies, may provide useful information on severity of illness and prognosis. As a collateral finding, the observed correlations were consistent with an impact of increased nutritional AA dose in moderating proteolysis.
